# Designing a heterostructure of graphite-like layer/TaO_x_ on polyetheretherketone for selective antibacteria and osteogenesis

**DOI:** 10.1093/rb/rbag152

**Published:** 2026-07-22

**Authors:** Shuaiqi Jiang, Junjie Zhou, Xue Ke, Yisi Liu, Xiang Pan, Dongrui Cai, Shiwei Guan, Ji Tan, Xuanyong Liu

**Affiliations:** State Key Laboratory of High Performance Ceramics, Shanghai Institute of Ceramics, Chinese Academy of Sciences, Shanghai 200050, China; Center of Materials Science and Optoelectronics Engineering, University of Chinese Academy of Sciences, Beijing 100049, China; School of Chemistry and Materials Science, Hangzhou Institute for Advanced Study, University of Chinese Academy of Sciences, Hangzhou 310024, China; State Key Laboratory of High Performance Ceramics, Shanghai Institute of Ceramics, Chinese Academy of Sciences, Shanghai 200050, China; State Key Laboratory of High Performance Ceramics, Shanghai Institute of Ceramics, Chinese Academy of Sciences, Shanghai 200050, China; Center of Materials Science and Optoelectronics Engineering, University of Chinese Academy of Sciences, Beijing 100049, China; State Key Laboratory of High Performance Ceramics, Shanghai Institute of Ceramics, Chinese Academy of Sciences, Shanghai 200050, China; Center of Materials Science and Optoelectronics Engineering, University of Chinese Academy of Sciences, Beijing 100049, China; Center of Materials Science and Optoelectronics Engineering, University of Chinese Academy of Sciences, Beijing 100049, China; School of Chemistry and Materials Science, Hangzhou Institute for Advanced Study, University of Chinese Academy of Sciences, Hangzhou 310024, China; Center of Materials Science and Optoelectronics Engineering, University of Chinese Academy of Sciences, Beijing 100049, China; School of Chemistry and Materials Science, Hangzhou Institute for Advanced Study, University of Chinese Academy of Sciences, Hangzhou 310024, China; State Key Laboratory of High Performance Ceramics, Shanghai Institute of Ceramics, Chinese Academy of Sciences, Shanghai 200050, China; State Key Laboratory of High Performance Ceramics, Shanghai Institute of Ceramics, Chinese Academy of Sciences, Shanghai 200050, China; State Key Laboratory of High Performance Ceramics, Shanghai Institute of Ceramics, Chinese Academy of Sciences, Shanghai 200050, China; School of Chemistry and Materials Science, Hangzhou Institute for Advanced Study, University of Chinese Academy of Sciences, Hangzhou 310024, China

**Keywords:** tantalum oxide, oxygen-vacancy, electron transfer, antibacterial, osteogenesis, polyetheretherketone

## Abstract

Implant-associated infection remains a critical challenge for orthopedic implants, particularly for bioinert polyetheretherketone (PEEK), which also requires improved osteogenic support. Here, we developed a heterostructured surface on PEEK by coupling a graphite-like layer with a defect-rich TaO_x_ nanoarray. The graphite-like layer promoted uniform TaO_x_ nanoarray formation and may facilitate interfacial electronic coupling, while the TaO_x_ nanoarray showed polarity-dependent and interface-limited charge-transport behavior in model electrical tests. Upon bacterial contact, the heterostructure produced a bacteria-associated 3,3′,5,5′-tetramethylbenzidine (TMB) oxidative response and markedly decreased bacterial ATP levels, leading to broad-spectrum antibacterial performance. Thermal annealing attenuated defect-associated TaO_x_ states and weakened antibacterial activity, supporting the involvement of defect-related interfacial states in the antibacterial process. Meanwhile, Ta/H-PEEK supported osteoblast proliferation and osteogenic differentiation *in vitro* and promoted peri-implant bone formation *in vivo*. The surface also reduced early bacterial burden in a rat infection model. Collectively, this work demonstrates a heterostructure-based PEEK surface strategy to integrate antibacterial activity with osteogenic compatibility.

## Graphical abstract

**Graphical Abstract:**
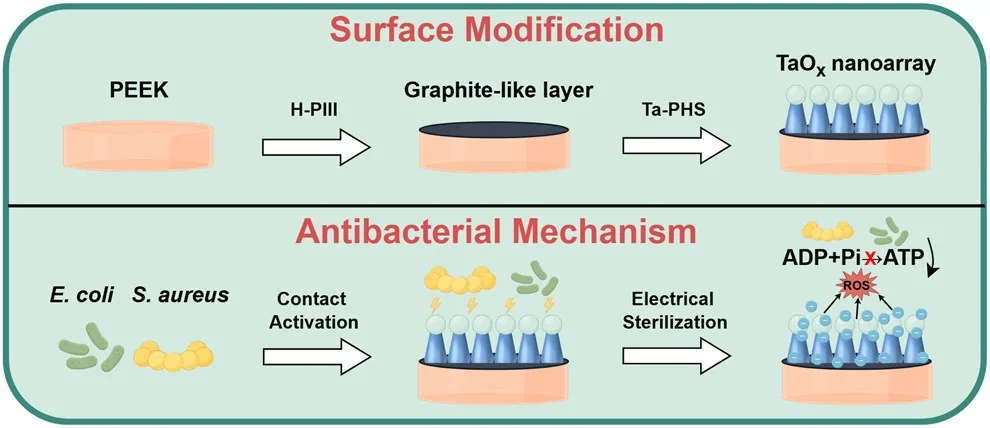

## Introduction

Implant-associated infection remains one of the most challenging complications in orthopedic surgery. In high-value applications such as critical-sized bone defect repair, fracture fixation, joint arthroplasty and spinal instrumentation, infection often results in implant failure and revision surgery and may progress to severe sequelae such as osteomyelitis [1–4]. An ideal orthopedic implant should therefore combine antibacterial capability with the ability to promote bone repair and osteogenesis. Polyetheretherketone (PEEK) has been regarded as an important alternative to metallic implants because of its elastic modulus close to that of cortical bone, corrosion resistance, radiolucency and excellent processability [5–7]. However, the chemical inertness of PEEK surfaces renders them susceptible to bacterial adhesion and biofilm formation *in vivo* while providing limited osteogenic support. Consequently, high infection risk and insufficient osteogenic support constitute two key barriers to its long-term clinical success. Constructing a functional interface on PEEK that simultaneously delivers efficient antibacterial activity and osteogenic support is therefore critical for improving implant safety and service life [8–10].

Current antibacterial strategies for implants mainly include antibiotic/drug-eluting coatings, metal-ion release systems, and contact-killing surfaces based on micro-/nano-topographies [11, 12]. These approaches are often accompanied by risks of antimicrobial resistance [13, 14], difficulty in maintaining long-term stable release kinetics [15, 16], and potential cytotoxicity or immune interference [17, 18]. More importantly, strong antibacterial activity does not necessarily translate into improved osseointegration and, in some cases, may even disrupt the osteogenic microenvironment [19, 20]. In recent years, an ‘electron-transfer interference’ paradigm has attracted increasing interest: by perturbing the bacterial membrane respiratory chain or extracellular electron transfer, battery-like interfaces, micro-galvanic effects and surface charge regulation can reduce the proton motive force, suppress ATP generation and induce membrane potential dysregulation, thereby enabling relatively stable bacteriostasis without relying on high-dose leaching or strong chemical oxidative stress [21–25]. Furthermore, if electron-transfer-related antibacterial effects can be coupled with the bioactivity of osteogenic elements, it may be possible to introduce osseointegration gains while establishing antibacterial protection, enabling an interface design that simultaneously addresses antibacterial and osteogenic requirements.

Tantalum (Ta) and its oxides have been widely used for surface modification of bone-contacting implants due to their excellent biocompatibility and consistently reported osteogenic/osseointegration performance [26]. Meanwhile, whether Ta or TaO_x_ exhibits stable and predictable antibacterial activity remains controversial [27]. Prior studies suggest that the antibacterial performance of Ta-based coatings may depend on the electrical coupling state between the coating and the substrate (e.g. electrochemical interfacial effects in metal-metal systems) [28, 29], yet a unified explanation is still lacking, particularly one that can be generalized across insulating versus conductive substrates [30]. Here, we adopt a new perspective: rather than treating Ta’s antibacterial behavior as an intrinsic material property, we view the native non-stoichiometric TaO_x_ shell as an electroactive interface whose behavior may be associated with defect-related states. In fields such as memristive devices, relaxation and migration of oxygen vacancies in TaO_x_ can reconstruct electron-transport pathways, and the interfacial barrier and electrode selection play decisive roles in charge transfer across metal/oxide interfaces [31–34]. This defect-enabled electrical tunability is instructive for biointerfaces: bacterial viability critically depends on membrane potential and respiratory electron transport, such that subtle perturbations in interfacial charge/electron processes may be amplified into metabolic imbalance and ATP depletion. Meanwhile, oxygen-vacancy-related states in defective oxides provide a material basis for non-antibiotic regulation of interfacial charge distribution and electrochemical behavior [35–37]. Notably, realizing a controllable electrically coupled interface on insulating polymers such as PEEK is nontrivial, highlighting the need for an addressable conductive ‘coupling base’. For example, a graphite-like conductive layer can be introduced via hydrogen plasma immersion ion implantation (H-PIII) to provide low-resistance transport and support the electrical response of a defect-rich oxide interface [38].

On this basis, we propose a mechanism-driven heterostructured interface in which a graphite-like conductive layer and a defect-rich TaO_x_ nanoarray are integrated on a PEEK substrate to form a ‘graphite-like layer/TaO_x_ nanoarray’ heterosystem. This design targets two coupled challenges. First, the graphite-like layer may serve as a conductive coupling interlayer and modulate Ta-based nanoarray nucleation, leading to improved uniformity and compactness. Second, defect-associated states in TaO_x_ provide electroactive interfacial features for exploring electron-transfer-related antibacterial effects within a controllable structural framework. This work further proposes and validates a testable working model for contact-associated antibacterial action and provides a surface strategy to achieve selective antibacterial activity and osteogenesis on inert polymer-based implants.

## Materials and methods

### Sample preparation

Biomedical-grade PEEK (Junhua Special Engineering Plastic Products, Jiangsu, China) was used to prepare all specimens for subsequent tests. The samples were machined into different geometries: discs (Φ 12 mm × 2 mm) for *in vitro* studies and discs (Φ 2 mm × 7 mm) for animal experiments. Prior to plasma treatments, all specimens were mechanically polished and then ultrasonically cleaned in acetone, absolute ethanol, and deionized water to remove surface contaminants. The cleaned specimens were dried in an oven and stored for further use.

H-PIII and Ta-PHS treatments were performed using a PBII-2 multifunctional composite surface modification system (CNNC Tongchuang Technology Co., Ltd., China). Specimens were placed in a vacuum chamber evacuated to ∼5 × 10^−3^ Pa. H-PIII was conducted using the key parameters listed in Table 1, and the resulting samples were denoted as H-PEEK. Subsequently, Ta deposition by plasma homopolar sputtering (PHS) was carried out on PEEK and H-PEEK using the key parameters listed in Table 2, yielding Ta-PEEK and Ta/H-PEEK, respectively. During both PIII and PHS processes, the sample holder was continuously rotated to ensure uniform implantation and deposition. High-purity Ar and H_2_ gases (99.999%, Shanghai Yujiali Special Gas Co., Ltd., China) were used.

**Table 1:** Main conditions for H-PIII.

Parameters	H-PEEK, Ta/H-PEEK
Gas flow of H_2_ (sccm)	30
Pressure (Pa)	2.0 × 10^−1^
Voltage (kV)	−30
Voltage pulse duration (μs)	30
Pulsing frequency (Hz)	100
RF power (W)	110
Time (min)	60

**Table 2:** Main conditions for Ta-PHS.

Parameters	Ta-PEEK, Ta/H-PEEK
Sputtering target	99.99% pure Ta
Gas flow of Ar (sccm)	30
Pressure (Pa)	7.5 × 10^−1^
Bias voltage (V)	800
Duty cycle (%)	30
RF power (W)	300
Time (min)	60

### Materials characterization

Surface morphologies were characterized by scanning electron microscopy (SEM; Sigma 300, Zeiss, Germany). Sample topography and roughness were measured by atomic force microscopy (AFM; Dimension XR, Bruker, Germany) in contact mode. Cross-sectional morphology and elemental distribution were examined using SEM (Sigma360, Zeiss, Germany) equipped with energy-dispersive spectroscopy (Xplore30, Oxford, UK). Crystalline phases were analyzed by X-ray diffraction (XRD; D/Max, Rigaku, Japan). Surface chemical states were evaluated by X-ray photoelectron spectroscopy (XPS; K-Alpha, Thermo Scientific, USA). Optical spectra were acquired using a UV–Vis–NIR spectrophotometer (Lambda 750, PerkinElmer, USA). Raman spectra were collected using a 514-nm excitation laser (HR800, HORIBA Jobin Yvon, France). Surface chemical functional groups were analyzed by Fourier-transform infrared spectroscopy (FTIR; FTIR-7600, Lambda Scientific, Australia). Zeta potential was measured using a SurPASS electrokinetic analyzer (Anton Paar, Austria). Electrical measurements were performed using a semiconductor parameter analyzer (B1500A, Agilent, USA). For two-probe I–V measurements, the distance between the two probes was 700 μm. Cyclic voltammetry (CV) was conducted using an electrochemical workstation (PGSTAT302N, Metrohm Autolab, Switzerland). Surface wettability was evaluated by the sessile drop method using distilled water and measured with a contact-angle goniometer (SL200B, Solon Information Technology Co., Ltd., China). To assess the time-dependent stability of surface wettability, Ta-PEEK and Ta/H-PEEK samples were stored in air for 14 days after preparation, and static water contact angles were measured on Days 1 and 14. To evaluate friction stability, a wear-resistance test was performed by placing each sample against sandpaper with 280# grit under a 200-g load. A sliding distance of 10 cm was defined as one cycle, and the sample weight before and after testing was recorded to calculate weight loss. To examine the effect of heat treatment on surface morphology and O 1s chemical states, Ta-PEEK and Ta/H-PEEK samples were treated at 100°C for 6 h, followed by SEM observation and O 1s XPS peak fitting.

### *In vitro* antibacterial performance evaluation

To evaluate antibacterial performance, Gram-negative *Escherichia coli* (*E. coli*, ATCC 25922) and Gram-positive *Staphylococcus aureus* (*S. aureus*, ATCC 25923) were used. Before bacterial culture, specimens were sterilized by immersion in 75% (v/v) ethanol for 2 h, with the ethanol solution replaced every 30 min. After sterilization, the samples were air-dried and placed in 24-well plates.

A 60-μL bacterial suspension (1 × 10^6^ CFU/mL) was dropped onto each sample surface and incubated at 37°C for 24 h. The bacterial suspension was then diluted 100-fold, and 100 μL of the diluted solution was spread onto agar plates and incubated at 37°C for 18 h. Agar plates were photographed, and colonies were counted. Antibacterial efficiency was calculated as follows:


(1) antibacterial rate=(A−B)/A×100%

*A* represents the number of colonies on PEEK in the control group, and *B* represents the number of colonies in the experimental group. For morphological observation of adherent bacteria, samples were fixed with 500 μL of 2.5% (v/v) glutaraldehyde, dehydrated through a graded ethanol series, and observed by field-emission SEM (FE-SEM; S-4800, HITACHI, Japan). A Tetramethylbenzidine (TMB) colorimetric assay was performed to evaluate the bacteria-associated interfacial oxidative response of the sample surfaces. For each group, two conditions were prepared: with bacteria and without bacteria. The total reaction volume was 500 μL. Specifically, 50 μL of HCl-TMB solution (200 mM) was added, followed by 350 μL of bacterial suspension (1 × 10^6^ CFU/mL in normal saline) for the bacteria group, or 350 μL of normal saline for the bacteria-free control. The remaining volume was adjusted to 500 μL with normal saline. The mixtures were incubated at 37°C for 24 h. After incubation, 100 μL of the reaction solution (supernatant) was collected and transferred to a 96-well plate, and the absorbance at 652 nm was measured using a microplate reader. TMB is oxidized into a blue-oxidized product with characteristic absorbance at 652 nm. An enhanced ATP assay kit (Beyotime, China) was used to quantify intracellular ATP levels of bacteria.

### *In vitro* osteogenic performance evaluation

Mouse pre-osteoblastic cells (MC3T3-E1) and rat bone marrow mesenchymal stem cells (rBMSCs) were used to evaluate osteogenic performance. Cells were cultured at 37°C in a humidified 5% CO_2_ incubator using α-MEM (Gibco, USA) supplemented with 10% fetal bovine serum (Gibco, USA) and 1% penicillin-streptomycin (Gibco, USA). The culture medium was refreshed every 3 days.

For cell adhesion observation, rBMSCs (2 × 10^4^ cells/mL) were seeded onto the sample surfaces. After 6 and 24 h of incubation, the medium was removed and samples were gently rinsed with PBS. Samples were dehydrated using graded ethanol, dried and observed by FE-SEM (S-4800, HITACHI, Japan).

Cell proliferation was assessed using the Alamar Blue assay (Alamar Blue™, Thermo Fisher Scientific, USA). Alamar Blue is a non-toxic blue dye that allows evaluation of cell viability without damaging cells. Briefly, 1 mL of MC3T3-E1 cell suspension (2 × 10^5^ cells/mL) was seeded onto each sample and cultured for 1, 4 and 7 days. At each time point, samples were gently rinsed twice with PBS and incubated with 500 μL medium containing 10% Alamar Blue for 2 h. Fluorescence intensity was measured with excitation at 560 nm and emission at 590 nm.

Alkaline phosphatase (ALP) expression was evaluated using a BCIP/NBT ALP staining kit (Beyotime, China). rBMSCs (1 × 10^4^ cells/mL) were seeded onto samples and cultured for 7 days. Cells were fixed with 4% paraformaldehyde and stained for 2 h. Images were acquired using bright-field microscopy (Olympus, Japan). For quantitative analysis, cells were incubated with p-nitrophenyl phosphate at 37°C for 0.5 h, and ALP activity was determined by measuring OD at 405 nm. Total protein (BSA equivalent) was determined using a BCA assay kit (Beyotime, China). ALP activity was normalized to the corresponding protein content.

Extracellular matrix mineralization was assessed by Alizarin Red S staining. rBMSCs (5 × 10^3^ cells/mL) were seeded on samples and cultured for 14 days. After culture, cells were rinsed with PBS, fixed with 75% ethanol, stained with 40 mM Alizarin Red S and washed repeatedly with ultrapure water. For quantification, the bound dye was dissolved using 10% cetylpyridinium chloride, and absorbance was measured at 620 nm.

Collagen secretion was evaluated by Sirius Red staining. rBMSCs (5 × 10^3^ cells/mL) were seeded on samples and cultured for 14 days. Cells were rinsed with PBS, fixed with 4% formaldehyde, stained with 0.1% Sirius Red and washed multiple times with 0.1 M acetic acid. For quantification, the dye was dissolved in a mixed solution of sodium hydroxide and methanol, and absorbance was measured at 540 nm.

### *In vivo* antibacterial performance evaluation

All animal experiments were approved by the Institutional Animal Care and Use Committee of SHRM (approval No. WM202408(27)) and were performed in accordance with the Animal Management Rules of the Ministry of Health of the People’s Republic of China and the Guidelines for the Care and Use of Laboratory Animals of China. A rat osteomyelitis model was established to evaluate the early antibacterial performance of the implants under an infected condition. Male Sprague–Dawley rats (220–250 g) were anesthetized by intraperitoneal injection of 10% pentobarbital sodium, followed by shaving and disinfection of the operative hindlimb. A medial parapatellar approach was used to expose the femoral condyle, and a 2-mm channel was drilled along the femoral axis into the medullary cavity.

To introduce bacterial contamination, the cylindrical implants (Φ 2 mm × 7 mm) were immersed in a *S. aureus* suspension (ATCC 25923, 1 × 10^6^ CFU/mL) prior to implantation. The bacteria-contaminated implants were then inserted into the medullary canal, and the incision was closed in layers. At 2 days post-surgery, the femurs with implants were harvested for antibacterial assessment.

To quantify bacterial burden on the retrieved implants, each implant was subjected to a standard roll-plate assay on agar plates to visualize viable bacteria, followed by plate counting after incubation. In addition, peri-implant tissues were collected for histological evaluation. Hematoxylin and eosin (H&E) staining was performed to assess inflammatory cell infiltration, and Giemsa staining was used to visualize bacteria in the infected tissues.

### *In vivo* osteogenic performance evaluation

A rat femoral implantation model was used to evaluate osteogenesis under non-infected conditions. Fourteen male Sprague–Dawley rats (220–250 g) were randomly assigned to two groups, namely PEEK and Ta/H-PEEK. Rats were anesthetized by intraperitoneal injection of 10% pentobarbital sodium. After shaving and disinfection of the hindlimb, a medial parapatellar incision was made to expose the femoral condyle. A 2-mm drill bit was used to create a channel along the femoral axis into the medullary cavity, and a cylindrical implant (Φ 2 mm × 7 mm) was inserted into the drilled hole. Femoral specimens were harvested at 6 weeks post-operation and analyzed by micro-computed tomography (micro-CT; IMAGING 100, Raycision Medical Technology Co., Ltd., China). Bone volume fraction (BV/TV) and bone mineral density (BMD) were calculated from the reconstructed datasets.

### Statistical analysis

GraphPad Prism 10.0 statistical software (GraphPad Software, CA, USA) was used to analyze quantitative data and display the data as mean ± standard deviation. Single-factor analysis of variance was used to determine the significance of differences. *P < 0.05, **P < 0.01 and ***P < 0.001 were considered statistically significant.

## Results and discussion

### Material preparation and characterization

**Figure 1:**
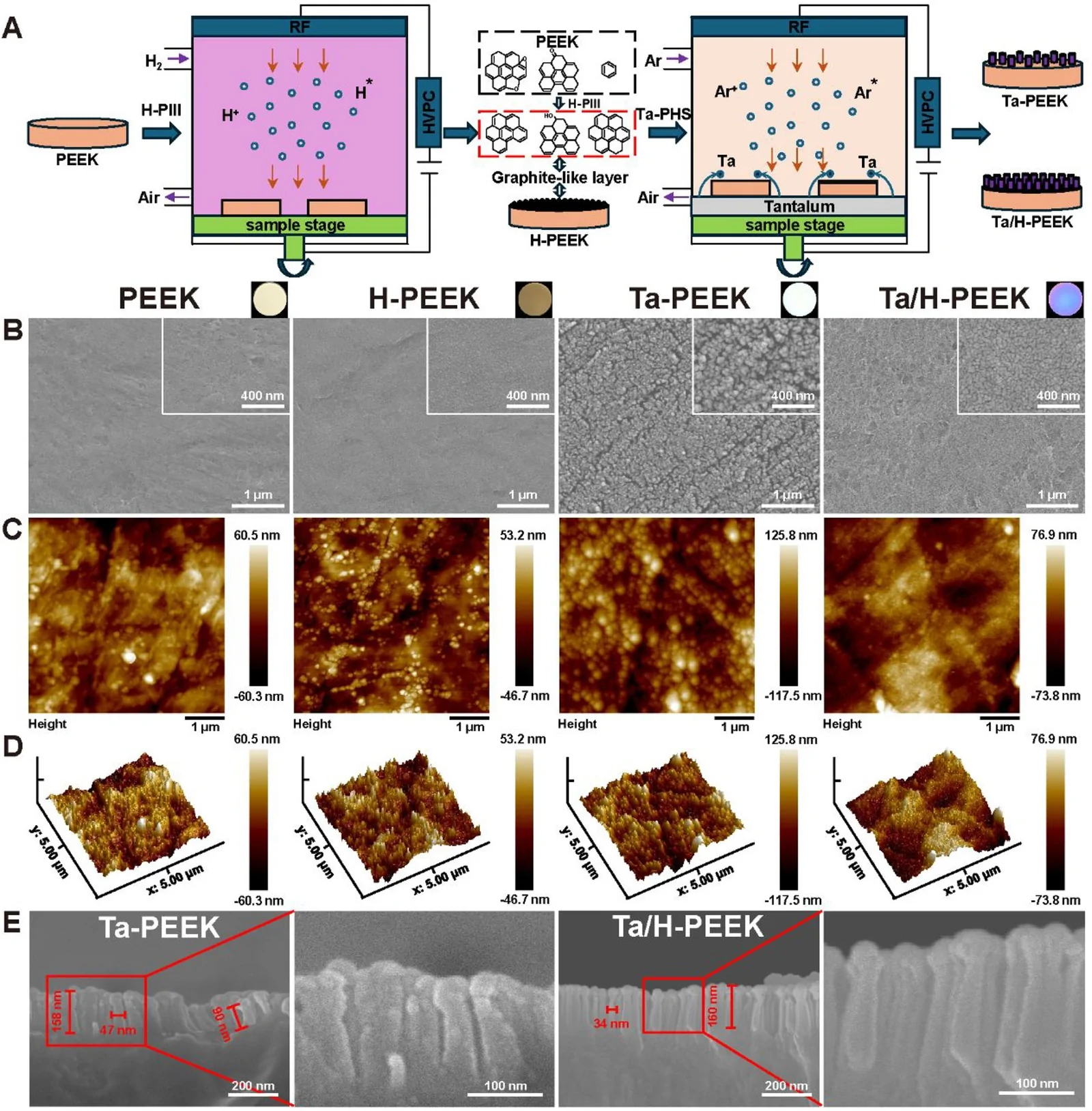
Fabrication and morphology of the designed heterostructure. (**A**) Schematic illustration of the two-step surface modification to generate Ta/H-PEEK. (**B**) SEM images, (**C**) 2D AFM images and (**D**) 3D AFM images of PEEK, H-PEEK, Ta-PEEK and Ta/H-PEEK. (**E**) SEM image of the Ta-PEEK and Ta/H-PEEK cross-sections.

As illustrated in Figure 1A, we constructed a designed interfacial architecture on PEEK via a two-step process. PEEK was first treated by H-PIII to generate H-PEEK with a graphite-like near-surface layer formed by carbonization/conjugation reconstruction. Ta nanostructures were then deposited on both PEEK and H-PEEK by PHS, yielding Ta-PEEK and Ta/H-PEEK.

Figure 1B presents representative SEM images and macroscopic photographs of the four groups. Polished PEEK exhibited a smooth and featureless surface. After H-PIII, H-PEEK remained largely smooth, with only sparse nanoscale dots, indicating that the implantation step introduced limited topographical changes at this length scale. Consistent with our previous observations, hydrogen species can remove oxygen-containing groups and promote an sp^2^-enriched carbon framework to form a graphite-like reconstruction. After Ta deposition, both Ta-PEEK and Ta/H-PEEK displayed abundant nanoscale surface features. Notably, Ta/H-PEEK showed a denser and more uniform distribution than Ta-PEEK, suggesting that the underlying graphite-like layer alters nucleation behavior (e.g. surface energy and nucleation density) and improves the uniformity of the TaO_x_ nanoarray.

AFM results (Figure 1C and D) further support the morphological differences. The Ra values were 17.4, 13.8, 33.7 and 22.7 nm for PEEK, H-PEEK, Ta-PEEK and Ta/H-PEEK, respectively. H-PEEK remained relatively smooth with only a few nanoscale dots. Ta-PEEK exhibited the highest roughness with pronounced granular protrusions and clear grain boundaries. In contrast, Ta/H-PEEK showed a more uniform nanostructure and a lower overall roughness than Ta-PEEK, consistent with the SEM observations.

SEM cross-section (Figure 1E) revealed ‘mushroom-like’ Ta nanopillar arrays on both Ta-PEEK and Ta/H-PEEK. This geometry is consistent with island-type (Volmer–Weber) nucleation and coalescence commonly observed for metal films on weakly wetting polymer substrates [39]. In Ta-PEEK, the pillar height and width were ∼90–155 nm and ∼ 35–50 nm, respectively, with poorer uniformity. In Ta/H-PEEK, the pillars were more regular and compact, with a height of ∼170 nm and a width of ∼ 35 nm. A top ‘cap’ layer with distinct contrast (thickness ∼45 nm) was observed, which is reasonably attributed to an oxide-enriched shell formed by oxidation of Ta upon exposure to air environments. This morphological feature is consistent with the ‘outer oxide/inner metal’ configuration later supported by XPS depth profiling. The inter-pillar gaps in Ta-PEEK may facilitate medium ingress and promote further oxidation, whereas the denser and more uniform top layer in Ta/H-PEEK may reduce penetration and thereby help maintain a more stable inner Ta core, providing a structural basis for the performance differences discussed below. To further evaluate the friction stability of the modified surfaces, a wear-resistance test was performed using sandpaper under a 200 g load (Supplementary Figure S1A). No significant difference in weight loss was observed among PEEK, H-PEEK, Ta-PEEK and Ta/H-PEEK (Supplementary Figure S1B), indicating that the graphite-like layer/TaO_x_ modification did not increase material loss under the tested friction condition. This result suggests that Ta/H-PEEK maintained comparable wear resistance to pristine PEEK in this assay.

XRD and XPS were used to examine whether the surface treatments introduced detectable phase changes and to clarify the near-surface chemical reconstruction. XRD patterns (Supplementary Figure S2) were dominated by the characteristic peaks of bulk PEEK, and no additional crystalline phases were detected. The reason is that the modified layer is only ∼170 nm; thus, signals from the surface layer can be largely masked by the substrate. In addition, the graphite-like layer and the TaO_x_ may be partially amorphous or poorly crystalline, further reducing the likelihood of detectable diffraction peaks. XPS survey spectra (Supplementary Figure S3) showed an increased C 1s signal and a decreased O 1s signal after H-PIII. After Ta-PHS, Ta signals emerged at ∼24 eV (Ta 4f), 237.6 eV (Ta 4d) and 400.6 eV (Ta 4p), confirming successful Ta deposition. High-resolution C 1s and O 1s spectra of PEEK and H-PEEK are shown in Figure 3A and B. The C 1s envelope was deconvoluted into sp^2^ carbon (C = C, 284.6 eV), sp^3^ carbon (C–C, 285.3 eV) and oxygen-containing components (C–O, C = O and O–C = O at 286.2, 286.8 and 288.4 eV). The O 1s envelope was fitted into the O–C, O–C = O and O = C components (531.3, 533.3 and 534.8 eV). A C–F-related contribution was also detected, consistent with the fluorinated units in PEEK. After H-PIII, the relative contributions of ether and carbonyl groups decreased, and the π–π* satellite feature associated with aromatic systems diminished/disappeared, indicating near-surface deoxygenation and chemical reconstruction changes consistent with the established capability of PIII to reconfigure polymer near-surface chemistry and related physicochemical properties.

**Figure 2:**
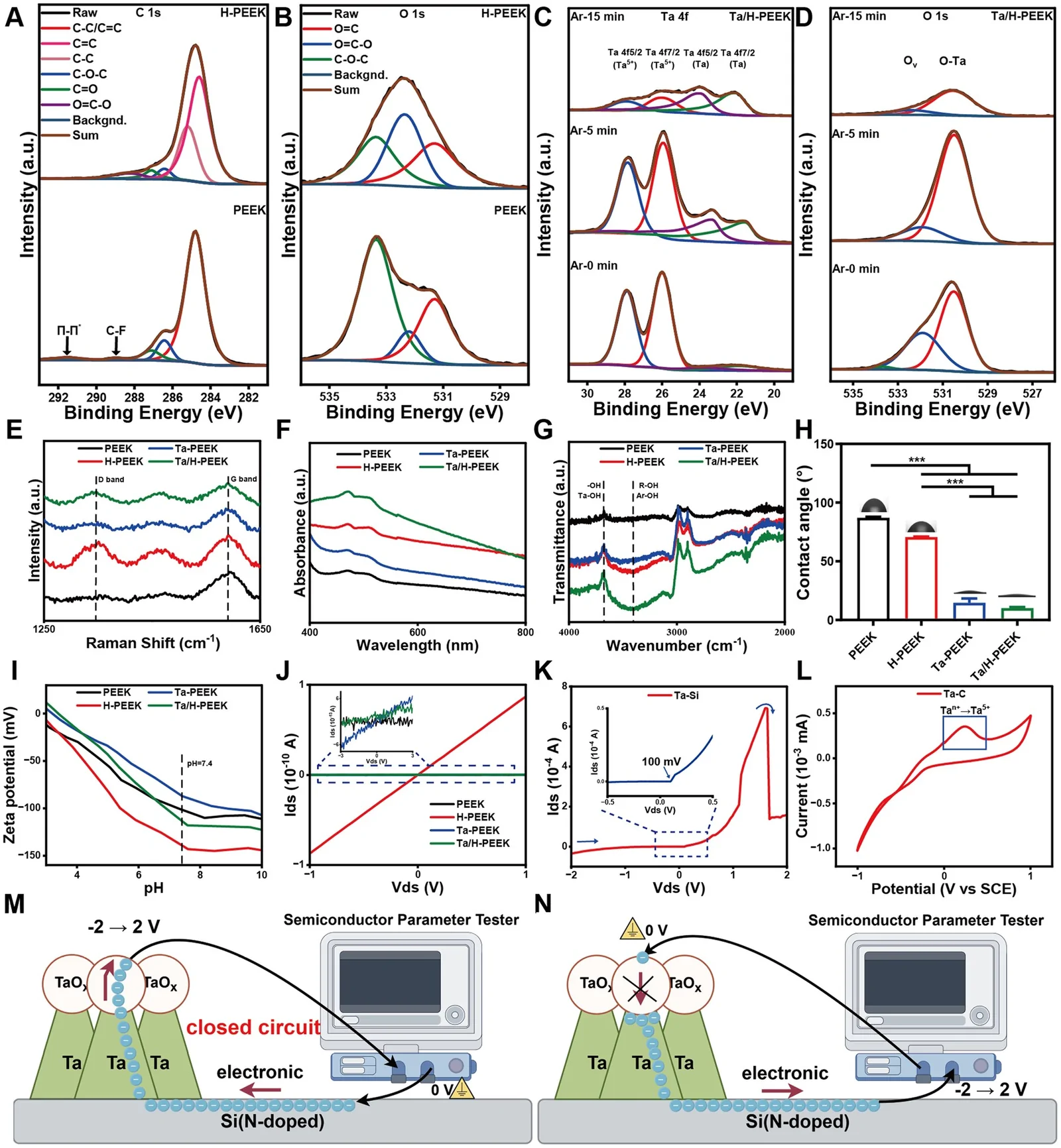
Physicochemical and electrical characterization. (**A** and **B**) High-resolution C 1s and O 1s spectra of PEEK and H-PEEK. (**C** and **D**) Ar-ion sputtering XPS depth profiles (Ta 4f and O 1s) of Ta/H-PEEK. (**E**) Raman spectra, (**F**) diffuse-reflectance UV–Vis spectra, (**G**) FTIR spectra, (**H**) static water contact angles of PEEK, H-PEEK, Ta-PEEK and Ta/H-PEEK measured 1 day after preparation, *n* = 3. (**I**) Zeta potential as a function of pH and (**J**) lateral two-probe I–V curves of the four groups. (**K**) Vertical I–V curves measured between an n-Si substrate and the nanopillar surface. (**L**) Cyclic voltammetry curves of the Ta-PHS layer deposited on a graphite carbon substrate. (**M** and **N**) Electron-flow schematics of Ta-Si. ***P < 0.001.

The XPS depth profiling was performed to resolve the depth-dependent Ta/TaO_x_ configuration and defect-related features. Ta readily forms a Ta(V)-dominated native oxide, and its oxidation/defect states can vary with depth. The sputtering rate was ∼2 nm/min, and Ta 4f and O 1s spectra were collected at 0, 5 and 15 min (corresponding to depths of 0, 10 and 30 nm). Ta 4f exhibits a spin-orbit doublet (Ta 4f7/2 and Ta 4f5/2). The Ta (0) doublet appears at 21.9 and 23.7 eV and may show asymmetry due to suboxides, whereas the Ta_2_O_5_ doublet appears at 26.0 and 27.9 eV. In O 1s, the component near 530.5 eV is assigned to lattice oxygen (O − Ta), the 532 eV component is commonly associated with vacancy/defect-related oxygen states in non-stoichiometric oxides, and the 533.5 eV component corresponds to surface-adsorbed species. As shown for Ta/H-PEEK in Figure 2C and D, the Ta (V) contribution decreased markedly with sputtering depth while the Ta (0) contribution increased, indicating an oxide-enriched outer region with a gradual transition to metallic Ta in the interior. For O 1s, the lattice-oxygen component increased first and then decreased, while the defect-related component gradually decreased; the adsorbed-oxygen signal was only observed at the top surface. Ta-PEEK exhibited a similar depth trend (Supplementary Figure S4A and B). Collectively, these results support hierarchical Ta nanopillar architecture comprising an outer TaO_x_ shell with depth-dependent defect-related features and an inner metallic Ta region, which provides the chemical/valence basis for the later discussion of electrically coupled interfacial processes.

Raman spectra (Figure 2E) showed a D band at 1345 cm^−1^ and a G band at 1585 cm^−1^. The D band reflects disordered/defective graphitic carbon, whereas the G band corresponds to vibrations of the sp^2^ carbon framework. Because PEEK contains aromatic units, all groups showed the G band. The D band became more pronounced after H-PIII, supporting the formation of a graphite-like reconstructed near-surface layer and aligning with the XPS evidence of deoxygenation and carbon-framework reconstruction.

Diffuse-reflectance UV–Vis spectra are shown in Figure 2F. Under this mode, the apparent absorbance reflects both intrinsic absorption and contributions from scattering/multiple reflections. In the visible range (400–800 nm), both H-PIII and Ta-PHS increased the apparent absorbance, and Ta/H-PEEK showed the strongest broadband response, consistent with a combined optical contribution from the graphite-like layer and the TaO_x_ nanoarray.

FTIR spectra (Figure 2G) showed broader –OH stretching bands (3600–3000 cm^−1^) on H-PEEK, Ta-PEEK and Ta/H-PEEK compared with PEEK, with the strongest signal on Ta/H-PEEK, indicating enriched hydroxyl/adsorbed-water-related species. A feature near ∼3680 cm^−1^ can be assigned to free − OH and isolated hydroxyl groups on TaO_x_ (Ta − OH). Consistently, the static water contact angle decreased from 87.1° for PEEK and 70.7° for H-PEEK to 14.6° for Ta-PEEK and 10.0° for Ta/H-PEEK, respectively, when measured 1 day after preparation (Figure 2H). These results indicate that the TaO_x_ nanoarray markedly enhanced the initial surface wettability of PEEK-based substrates. Considering possible time-dependent hydrophobic recovery, the wettability of Ta-PEEK and Ta/H-PEEK was further evaluated after 14 days of storage. Although the contact angles increased to 50.6° and 54.1°, respectively, both surfaces remained hydrophilic (Supplementary Figure S5).

We evaluated the interfacial charge state and charge-transport characteristics using zeta potential measurements and electrical testing. Figure 2I shows the pH-dependent zeta potential profiles of different samples. With increasing pH, the zeta potential of all groups shifts toward more negative values, indicating enhanced surface deprotonation. Under physiologically relevant conditions (pH 7.4), all surfaces are negatively charged; H-PEEK exhibits the most negative potential, followed by Ta/H-PEEK, whereas PEEK and Ta-PEEK show comparatively smaller negative values. These results indicate that H-PIII markedly modulates the interfacial charge microenvironment, and that the Ta-based nanoarray further influences the surface charge state. Given that bacterial envelopes are typically negatively charged, these differences are more likely to affect electrostatic interactions and the adhesion energy barrier during the initial bacteria-material contact, rather than independently accounting for the subsequent charge-transfer processes and metabolic-inhibition phenotypes.

To identify potential charge-transport pathways, we performed I–V measurements in both the in-plane and out-of-plane directions. In the in-plane two-probe I–V curves (Figure 2J), H-PEEK showed a much higher lateral conductance than the other three groups; therefore, the I–V curves of PEEK, Ta-PEEK and Ta/H-PEEK appeared compressed and partially overlapped when plotted together with H-PEEK on the same scale. To provide a quantitative comparison, surface conductivity was calculated from the lateral I–V curves (Supplementary Table S1). H-PEEK exhibited the highest surface conductivity, whereas PEEK, Ta-PEEK and Ta/H-PEEK were several orders of magnitude lower. The limited lateral conductivity of the TaO_x_-containing samples is attributed to the discontinuity between adjacent TaO_x_ nanostructures and the oxide nature of TaO_x_, which restricts lateral electron percolation across the surface. In contrast, H-PEEK displays an approximately linear I–V response from −3 to 3 V, confirming that the graphite-like layer induced by H-PIII substantially enhances in-plane conductivity. Notably, because the two probes are positioned on the same surface plane, this configuration primarily reflects lateral transport and does not directly capture vertical conduction along the nanoarray height.

Accordingly, we further constructed a vertical test configuration to evaluate charge transport across the nanoarray layer and along the substrate-normal direction. Because the PEEK-based substrates showed limited conductivity, direct vertical electrical testing on PEEK could not reliably reflect the electronic characteristics of the TaO_x_ layer. Therefore, an n-type Si substrate was used as a conductive/semiconductive model substrate to provide a stable bottom electrode and to resolve the vertical charge-transport behavior associated with the TaO_x_-containing interfacial structure. Figure 2K presents the vertical I–V curve acquired under a unidirectional voltage sweep (−2 → 2 V), where the n-type Si substrate is grounded and a bias is applied to the top of the TaO_x_ nanoarray. The current exhibits a pronounced nonlinear dependence on bias. In the low-bias regime, when the voltage increases to ∼ 0.1 V, the current rises abruptly by approximately two orders of magnitude (from ∼10^−6^ A to ∼10^−4^ A). With further increases in bias, the current continues to increase but remains non-ohmic. When the bias approaches ∼1.6 V, the current drops sharply, resembling the state-transition behavior commonly observed in defect-rich oxide systems. Moreover, upon reversing the electrode configuration (bias applied to the Si side and the TaO_x_ side grounded; Supplementary Figure S6), the current is strongly suppressed to ∼10^−11^ A with substantial fluctuations, overall approaching an insulating response. Schematics in Figures 2M and N illustrate the corresponding electron-flow directions under these two wiring modes. Collectively, these results demonstrate strong polarity dependence and interface-limited vertical transport in the TaO_x_/electrode stack, along with an excitable, state-dependent conduction behavior. This provides key electrical evidence to support subsequent discussion of defect-state-mediated interfacial charge/electron processes.

Because bulk PEEK is electrically insulating and the graphite-like conductive layer is confined to a near-surface region, direct CV testing on PEEK substrates could not provide a closed and stable electrical circuit. Therefore, a conductive graphite carbon substrate was used as a model substrate to probe the electrochemical propensity associated with the Ta-PHS-derived interfacial layer. To probe the electrochemical propensity associated with the ‘graphite-like layer/TaO_x_’ heterostructure, we deposited Ta-PHS on a conductive graphite carbon substrate to fabricate a model electrode (Figure 2L). Within the selected potential window, the CV curve exhibits a distinct anodic oxidation peak without a symmetric cathodic reduction peak, indicating an irreversible or quasi-irreversible process. This behavior is consistent with Ta surface oxidation and the formation of a passivating oxide film with kinetic hindrance, suggesting that the Ta/TaO_x_ interface possesses a potential-tunable oxidation tendency and the capacity for valence-state modulation. These findings provide complementary evidence supporting TaO_x_ as an electroactive interfacial unit.

### *In vitro* antibacterial performance and mechanism

**Figure 3:**
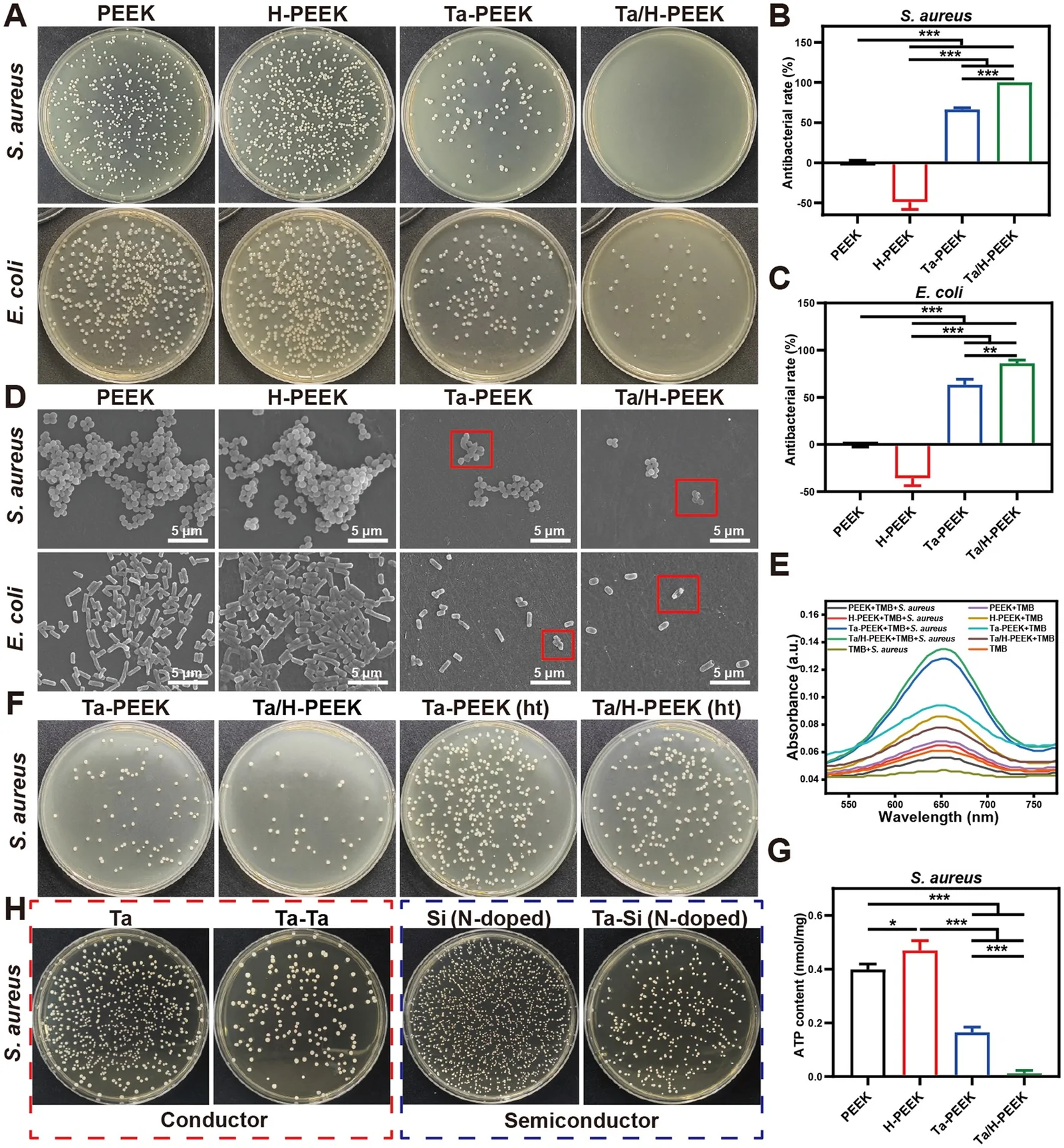
*In vitro* antibacterial evaluation. (**A**) Agar plate images of *S. aureus* and *E. coli* after incubation on different samples. (**B** and **C**) Antibacterial rates for *S. aureus* and *E. coli*, *n* = 3. (**D**) SEM images of bacteria adhered to sample surfaces. (**E**) TMB assay for assessing bacteria-dependent oxidative response after co-incubation with samples (with and without bacteria). (**F**) Agar plate images after heat treatment (ht) of Ta-PEEK and Ta/H-PEEK. (**G**) Intracellular ATP levels of *S. aureus* after incubation on different samples, *n* = 3. (**H**) Agar plate images for antibacterial comparison on different substrates (Ta, Ta-Ta, Si (N-doped) and Ta-Si (N-doped)). Alamar Blue fluorescence signals for bacterial viability/proliferation. *P < 0.05, **P < 0.01, ***P < 0.001.

*In vitro* antibacterial performance was evaluated using *S. aureus* and *E. coli* (Figure 3). Agar plate assays (Figure 3A) demonstrated a higher colony burden on H-PEEK compared to pristine PEEK, indicating that under the current conditions, the graphite-like conductive layer induced by H-PIII does not possess intrinsic bactericidal activity and may even reduce the initial adhesion barrier. In contrast, after introducing the Ta-based nanoarray, colony formation by both strains was markedly suppressed, with Ta/H-PEEK showing the strongest inhibition. Quantitative colony counting further corroborated these trends (Figure 3B and C). For *S. aureus*, the antibacterial rates of Ta-PEEK and Ta/H-PEEK were 66.46% and 100%, respectively; for *E. coli*, they were 63.46% and 86.06%, respectively. Notably, H-PEEK exhibited a negative ‘antibacterial rate’, further demonstrating that enhanced conductivity alone is not a sufficient determinant of the antibacterial phenotype. Instead, the antibacterial enhancement coincided more closely with the introduction of the Ta-based nanoarray featuring an oxide-rich (TaO_x_) outer shell, suggesting that this interfacial unit plays a critical role in antibacterial action. SEM observations of surface-adhered bacteria (Figure 3D) showed dense adhesion and aggregation on PEEK and H-PEEK, with bacteria maintaining typical intact coccoid or rod-like morphology. In contrast, substantially fewer bacteria were observed on Ta-PEEK and Ta/H-PEEK, and the remaining cells exhibited visible damage (e.g. wrinkling and collapse; marked regions), consistent with reduced colonization capacity.

To further probe whether the antibacterial phenotype is accompanied by activation of an interfacial oxidative response, we used TMB chromogenic conversion as a functional readout (Figure 3E). In the absence of exogenous hydrogen peroxide, Ta/TaO_x_-related samples exhibited a higher absorbance response at 652 nm in the presence of bacteria, whereas this enhancement was absent or markedly attenuated in bacteria-free controls. This bacteria-dependent triggering pattern suggests that bacterial contact may induce or amplify interfacial oxidative activity at the TaO_x_-containing interface, as reflected by TMB oxidation. Because TMB oxidation does not directly quantify ROS concentration and may also be influenced by bacterial metabolites, this result is interpreted as a supporting colorimetric indicator of bacteria-associated interfacial oxidative activity rather than a direct measurement of oxidative species.

Consistent with this triggered readout, intracellular ATP measurements for *S. aureus* (Figure 3G) showed comparable ATP levels on PEEK and H-PEEK, whereas ATP decreased significantly after incorporation of the Ta-based nanoarray. Ta/H-PEEK reduced ATP to near-background levels, indicating that the antibacterial effect is unlikely to arise solely from reduced adhesion and is more plausibly associated with suppression of bacterial energy metabolism.

We next introduced a mild heat-treatment control (ht; 100°C, 6 h) to perturb defect-/hydroxyl-related surface states within the TaO_x_ shell while avoiding severe thermal deformation of the PEEK substrate or collapse of the TaO_x_ nanoarray. After heat treatment, colony counts increased markedly (Figure 3F), and viability/proliferation-related readouts were substantially enhanced and approached control levels (Supplementary Figure S8). Additional SEM observation and O 1s XPS fitting showed that the TaO_x_ nano-coating was generally retained after heat treatment, without obvious cracking, delamination, or collapse, although slight enlargement/coarsening of some nanoparticles was observed (Supplementary Figure S7). O 1s XPS fitting further showed a decrease in the defect-associated oxygen component after heat treatment, supporting attenuation of defect-related TaO_x_ states. Therefore, the weakened antibacterial activity after heat treatment is associated with changes in defect-related TaO_x_ states and surface chemistry, while possible contributions from limited nanoscale morphological changes cannot be fully excluded.

Finally, to assess the applicability of the Ta-PHS-derived nanostructure across different electrical substrates (Figure 3H), we deposited the Ta-based nanostructure onto conductive and semiconducting substrates. In both cases, significant suppression of colony formation was observed, indicating a degree of substrate adaptability. For insulating polymer systems, we further found that introducing a graphite-like conductive layer prior to Ta deposition provided additional antibacterial enhancement.

This trend was also validated on another insulating polymer (PP) (Supplementary Figure S9): the H-PP substrate increased bacterial burden, Ta-PP showed only limited inhibition, whereas Ta/H-PP markedly reduced colony numbers. Taken together, results from plate counting, bacteria-triggered TMB readout, ATP depletion assays and defect-sensitivity controls converge on the same conclusion. These data support a mechanism in which a defect-active TaO_x_ shell is electrically coupled to the substrate through an underlying graphite-like layer. Upon bacterial contact, this coupled interface is proposed to initiate an interfacial oxidative response that suppresses the energy metabolism of adherent bacteria and yields a stable antibacterial phenotype.

**Figure 4:**
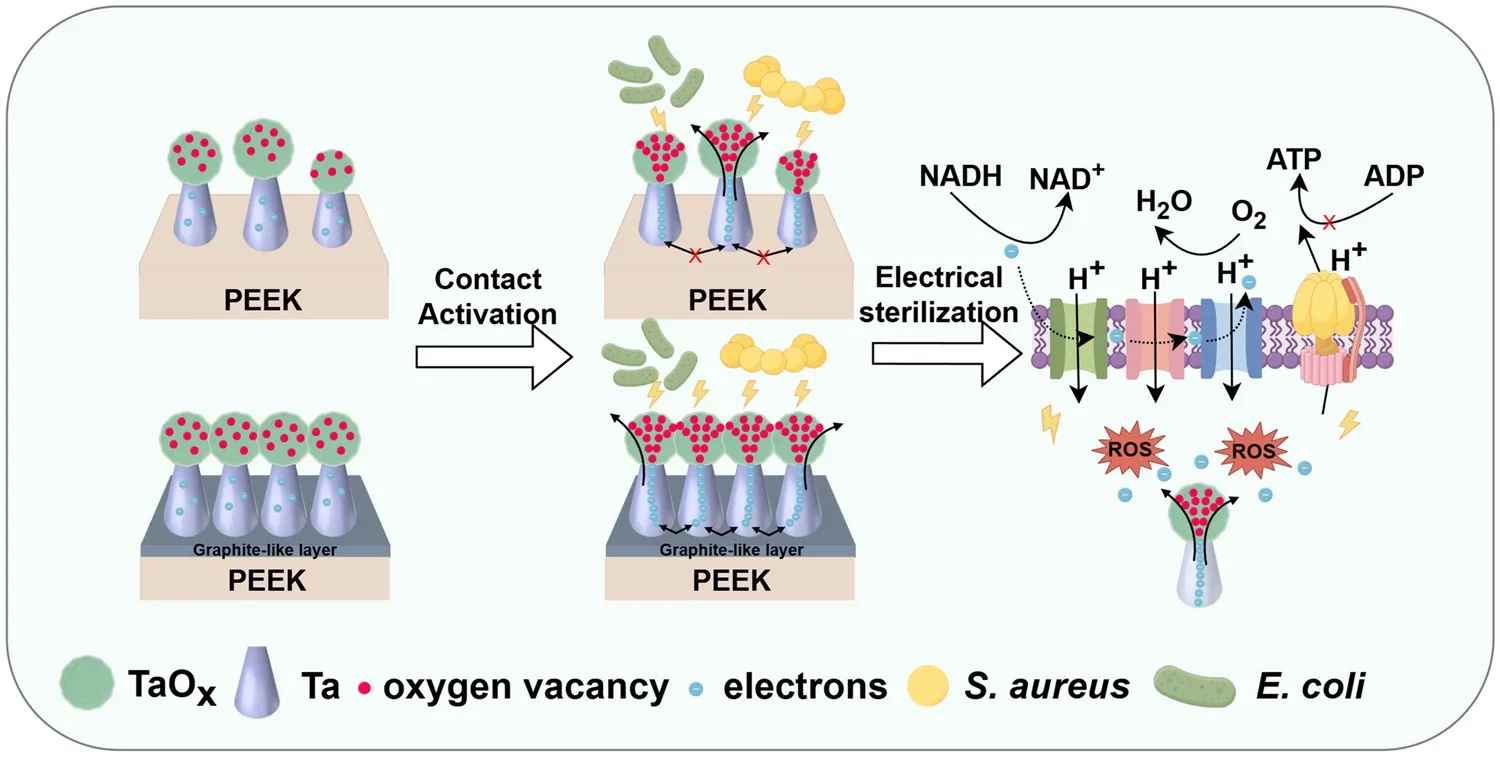
Antibacterial mechanism of the graphite-like layer/TaO_x_ via bacteria-triggered, defect-enabled electron-transfer interference.

Collectively, our results support a ‘bacteria-triggered, defect-enabled electron-transfer interference mechanism’ for the Ta/H-PEEK heterostructure (Figure 4). In the quiescent state, oxygen vacancies in the non-stoichiometric TaO_x_ shell undergo largely random relaxation, leading to an interface-limited, low-probability transport pathway for electrons from the Ta core to the outer surface. Upon bacterial adhesion, the local interfacial potential perturbs the defect landscape and biases vacancy relaxation/reconstruction, which increases the electron-transmission probability across the TaO_x_ shell. Importantly, the underlying graphite-like layer may act as a conductive coupling interlayer that facilitates charge communication with the TaO_x_ nanoarray and, indirectly, modulates nanoarray nucleation, interfacial bonding/stress and defect-related TaO_x_ states. The resulting interfacial charge/oxidative response may promote bacteria-localized oxidative stress at the bacteria-material interface, leading to membrane potential dysregulation, respiratory-chain impairment and pronounced ATP depletion, ultimately suppressing bacterial viability. Notably, the model emphasizes the defect-dependent and adhesion-triggered nature of the interfacial response, offering a unified explanation for our observed antibacterial trends (Figure 3).

### *In vitro* and *in vivo* biological activity

**Figure 5:**
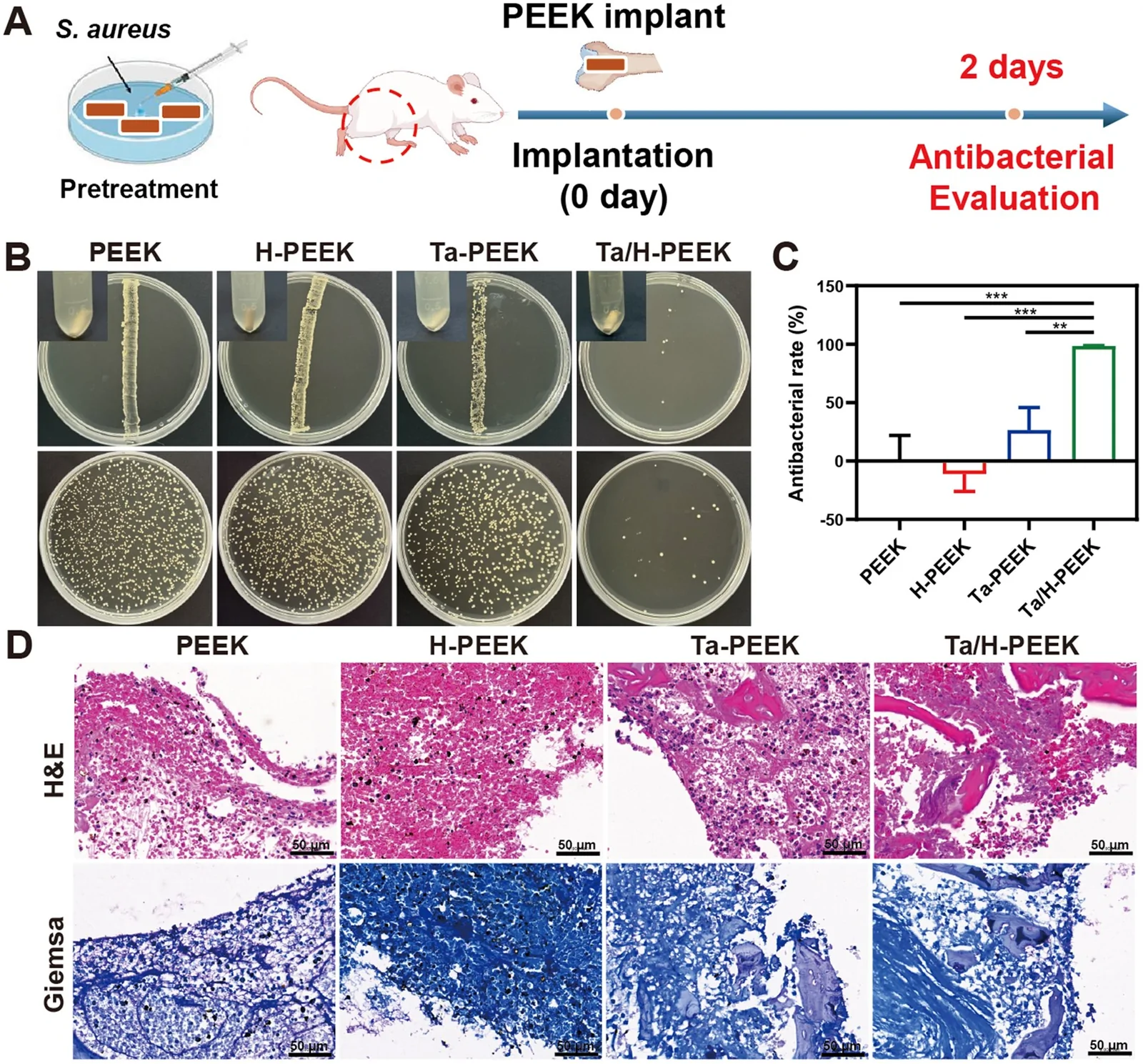
*In vivo* antibacterial evaluation in a rat osteomyelitis model. (**A**) Schematic of the implantation and infection model. (**B**) Rolling/printing and plate culture images of explanted implants at 2 days post-surgery. (**C**) Quantification of bacterial burden and calculated antibacterial rate, *n* = 3. (**D**) Histological images (H&E and Giemsa staining). **P < 0.01, ***P < 0.001.

Early *in vivo* antibacterial performance was evaluated in a rat osteomyelitis model challenged with *S. aureus*, and implants were retrieved 2 days postoperatively (Figure 5A). This early time point focuses on initial bacterial adhesion/colonization on implant surfaces and minimizes confounding contributions from later systemic inflammation and tissue remodeling.

Rolling/printing and plate cultures (Figure 5B) showed dense bacterial growth for PEEK and H-PEEK, indicating substantial early colonization. Ta-PEEK reduced colony formation but still showed notable residual bacteria. In contrast, Ta/H-PEEK exhibited a marked reduction in both rolling and plate colonies. Quantification and antibacterial-rate calculations (Figure 5C) confirmed that Ta/H-PEEK outperformed the other groups, demonstrating effective early suppression of implant-associated bacterial burden *in vivo*.

Histological analyses further supported these findings (Figure 5D). H&E staining revealed stronger inflammatory cell infiltration around PEEK and H-PEEK, partial alleviation for Ta-PEEK and the mildest inflammatory response for Ta/H-PEEK. Giemsa staining showed abundant bacteria-associated signals in PEEK and H-PEEK, whereas Ta/H-PEEK displayed markedly reduced bacterial signals, consistent with the microbiological results. Overall, Figure 5 demonstrates that the TaO_x_ nanopillar interface reduced early implant-associated bacterial burden and alleviated local inflammation at 2 days post-surgery. It should be noted that this short-term model does not assess mature biofilm formation, chronic osteomyelitis progression or long-term anti-infection efficacy; therefore, the *in vivo* antibacterial result should be interpreted as evidence of reduced early bacterial colonization/burden.

**Figure 6:**
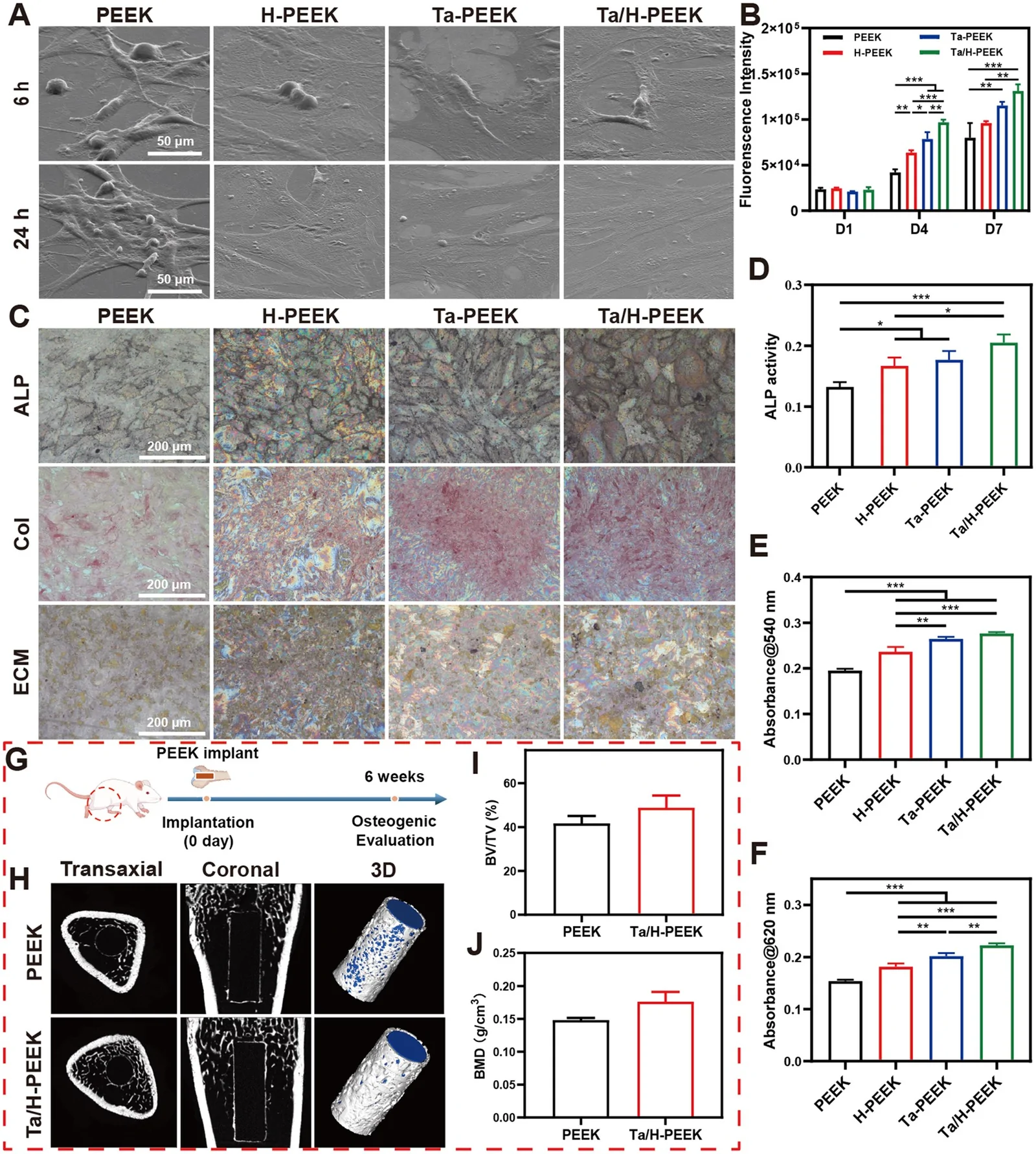
*In vitro* osteogenic evaluation and *in vivo* bone regeneration. (**A**) SEM images of rBMSCs adhesion on sample surfaces at 6 and 24 h. (**B**) MC3T3-E1 proliferation at Days 1, 4 and 7 (Alamar Blue), *n* = 3. (**C**) ALP staining (Day 7), collagen staining (Day 14) and mineralization staining (Day 14). (**D**) Quantification of ALP activity, *n* = 3. (**E**) Quantification of collagen deposition, *n* = 3. (**F**) Quantification of mineralization, *n* = 3. (**G**) Schematic of the rat femoral defect implantation model. (**H**) Micro-CT reconstructions at 6 weeks. (**I** and **J**) Quantitative micro-CT analysis (BV/TV and BMD), *n* = 3. *P < 0.05, **P < 0.01, ***P < 0.001.

For bone-repair applications with infection risk, antibacterial function should be achieved without sacrificing osteogenic support (Figure 6). rBMSCs adhesion SEM images (Figure 6A) showed that cells attached and spread on all surfaces at 6 and 24 h, forming typical pseudopodia. The modified groups (H-PEEK, Ta-PEEK and Ta/H-PEEK) exhibited more extensive spreading, suggesting improved early cell-surface interactions.

MC3T3-E1 proliferation (Figure 6B) increased over time on all samples, with an overall trend of Ta/H-PEEK > Ta-PEEK > H-PEEK > PEEK, indicating enhanced cytocompatibility/bioactivity after modification. Consistently, ALP staining and quantification at Day 7 (Figure 6C and D) were higher in the modified groups, suggesting promoted early osteogenic differentiation. At Day 14, collagen deposition and mineralization (Figure 6C, E and F) were also enhanced, with Ta/H-PEEK showing the strongest effect. Since orthopedic implants inevitably contact blood during implantation, hemolysis testing was performed to evaluate blood compatibility. As shown in Supplementary Figure S10, the positive control showed obvious hemolysis, whereas the negative control and all sample groups showed clear supernatants after centrifugation. Compared with the negative control, PEEK, H-PEEK, Ta-PEEK and Ta/H-PEEK showed lower hemolysis ratios, indicating that the modified PEEK surfaces exhibited good blood compatibility under the tested conditions.

*In vivo* bone regeneration was further assessed in a rat femoral defect model (Figure 6G). Micro-CT reconstructions at 6 weeks (Figure 6H) revealed more newly formed bone in the defect/implant-associated region for Ta/H-PEEK. Quantitative metrics (Figure 6I and J) showed higher BV/TV and BMD for Ta/H-PEEK than for PEEK, supporting improved peri-implant bone formation and mineralization. Together, these results demonstrate that the designed TaO_x_ nanopillar interface is compatible with osteogenesis and can promote osteogenic outcomes while providing antibacterial functionality.

## Conclusion

A heterostructure of a graphite-like conductive coupling layer and a defect-rich TaO_x_ nanoarray was engineered on PEEK. This architecture improved surface hydrophilicity, promoted uniform TaO_x_ nanoarray formation and provided electron-transfer-related interfacial features in model electrical tests. Functionally, Ta/H-PEEK delivered broad-spectrum antibacterial activity accompanied by bacterial ATP depletion, and its antibacterial performance was associated with defect-related TaO_x_ states and a bacteria-associated interfacial oxidative response. Importantly, the same interface supported osteogenic activity *in vitro* and promoted peri-implant bone formation *in vivo*, while reducing early bacterial burden in an infected animal model. Overall, this heterostructured PEEK surface provides a strategy to integrate antibacterial activity with osteogenic compatibility without relying on conventional release-based antibacterial modification.

## Supplementary Material

rbag152_Supplementary_Data
