# Supplementary material for: Designing a heterostructure of graphite-like layer/TaOx on polyetheretherketone for selective antibacteria and osteogenesis
Source: Regen Biomater. 2026 Jul 22;13:rbag152. doi: 10.1093/rb/rbag152 (PMC13548786; doi:10.1093/rb/rbag152)
Supplement: Supplementary file 1 [file rbag152_supplementary_data.docx]

Designing a Heterostructure of Graphite-like Layer/TaO_x_ on Polyetheretherketone for Selective Antibacteria and Osteogenesis

Shuaiqi Jiang ^a, b, c, 1^, Junjie Zhou ^a, 1^, Xue Ke ^a, b^, Yisi Liu ^a, b^, Xiang Pan ^b, c^, Dongrui Cai ^b, c^, Shiwei Guan ^a, *^, Ji Tan ^a, *^, and Xuanyong Liu ^a, c, *^

^a^State Key Laboratory of High Performance Ceramics, Shanghai Institute of Ceramics, Chinese Academy of Sciences, Shanghai 200050, China.

^b^Center of Materials Science and Optoelectronics Engineering, University of Chinese Academy of Sciences, Beijing 100049, China.

^c^School of Chemistry and Materials Science, Hangzhou Institute for Advanced Study, University of Chinese Academy of Sciences, Hangzhou 310024, China.

^1^These authors contributed equally to this work.

^*^Corresponding authors:

Xuanyong Liu, xyliu@mail.sic.ac.cn; Ji Tan, tanji@mail.sic.ac.cn; Shiwei Guan, guansw@hku.hk

**Figure S1**. Wear-resistance test of PEEK, H-PEEK, Ta-PEEK, and Ta/H-PEEK. (A) Schematic illustration of the wear-resistance test. (B) Weight loss of different samples after testing, n = 3.

Figure S1 shows the wear-resistance test used to evaluate the friction stability of different PEEK-based surfaces. No significant difference in weight loss was observed among PEEK, H-PEEK, Ta-PEEK, and Ta/H-PEEK, indicating that the graphite-like layer/TaO_x_ modification did not increase material loss under the tested condition.

**Figure S2.** XRD spectra of PEEK, H-PEEK, Ta-PEEK, and Ta/H-PEEK.

Figure S2 shows the X-ray diffraction (XRD) patterns of PEEK, H-PEEK, Ta-PEEK, and Ta/H-PEEK. All groups display the characteristic diffraction peaks of semicrystalline PEEK with comparable peak positions and overall profiles. No distinct additional diffraction peaks attributable to new crystalline inorganic phases are clearly resolved after H-PIII treatment or after introducing the Ta-based nanoarray, indicating that the bulk crystalline structure of the polymer substrate remains dominant in the diffractograms and that any newly formed surface layer contributes minimally to the XRD signal.

**Figure S3.** XPS survey spectra of PEEK, H-PEEK, Ta-PEEK, and Ta/H-PEEK.

Figure S3 presents the XPS survey spectra of PEEK, H-PEEK, Ta-PEEK, and Ta/H-PEEK, providing an overview of the near-surface elemental composition. The spectra of PEEK and H-PEEK are mainly composed of C 1s and O 1s signals, while the Ta-containing groups exhibit clear Ta-related peaks (e.g., Ta 4f, Ta 4d, Ta 4p regions in the survey scan), confirming successful incorporation of Ta-based species at the surface after Ta-PHS deposition. By comparing the relative intensity changes in the C 1s and O 1s signals among PEEK and H-PEEK, the survey scan also reflects the compositional shift induced by the plasma treatment at the outermost surface.

**Figure S4**. (A) Ta 4f spectra of Ta-PEEK at different Ar-ion sputtering times. (B) O 1s spectra of Ta-PEEK at different Ar-ion sputtering times.

Figure S4 shows the Ar-ion sputtering XPS depth profiles for Ta-PEEK, focusing on the evolution of Ta 4f and O 1s signals with sputtering time. As sputtering proceeds (probing progressively deeper regions), the Ta 4f spectral envelope changes, reflecting variation in the relative contributions of oxidized Ta components versus more metallic Ta components across the depth of the deposited layer. In parallel, the O 1s signal and its component distribution evolve with depth, indicating a non-uniform oxygen environment through the nanopillar coating. Overall, the depth profiling provides a depth-resolved chemical view of the Ta-based layer rather than a single surface snapshot.

**Figure S5**. Water contact angles of Ta-PEEK and Ta/H-PEEK measured on day 1 and day 14 after storage in air, n = 3.

Figure S5 shows the time-dependent water contact angle changes of Ta-PEEK and Ta/H-PEEK after storage in air. Although the contact angles increased after 14 days, both surfaces remained hydrophilic, indicating partial retention of the TaO_x_-induced hydrophilicity.

**Figure S6**. Vertical I-V curves measured between the substrate and nanopillar surface under reversed grounding/bias configuration.

Figure S6 presents the vertical current-voltage (I-V) curves measured under a reversed wiring configuration (reversed grounding/bias compared with the primary measurement setup), highlighting how the out-of-plane electrical response depends on the measurement polarity. The curves exhibit a pronounced polarity-dependent behavior in the current response, and the current level is markedly altered relative to the “forward” configuration reported in the main text. These data are included to document the robustness of the observed vertical transport characteristics under an alternative electrical connection scheme and to visualize the direction-sensitive electrical response of the TaO_x_ nanoarray interface.

**Figure S7**. Surface morphology and O 1s chemical states before and after heat treatment. (A) SEM images of Ta-PEEK, Ta-PEEK(ht), Ta/H-PEEK, and Ta/H-PEEK(ht). O 1s XPS spectra and peak fitting of Ta/H-PEEK (B) and Ta/H-PEEK(ht) (C) after Ar-ion sputtering for 0, 5, and 15 min.

Figure S7 shows the morphology and O 1s chemical-state changes before and after heat treatment. SEM images indicate that the TaO_x_ nano-coating was generally retained after heat treatment, with no obvious cracking, delamination, or collapse, although limited nanoparticle coarsening was observed. O 1s XPS fitting shows a decrease in the defect-associated oxygen component after heat treatment, supporting attenuation of defect-related TaO_x_ states.

**Table S1. Surface conductivity calculated from lateral I-V measurements.**

| Samples | Surface conductivity / S · mm^−1^ |
| --- | --- |
| PEEK | 7.72 × 10^−15^ |
| H-PEEK | 1.24 × 10^−10^ |
| Ta-PEEK | 2.93 × 10^−13^ |
| Ta/H-PEEK | 1.01 × 10^−13^ |

Table S1 summarizes the surface conductivities calculated from the in-plane two-probe I-V curves using a probe spacing of 700 μm. H-PEEK exhibited markedly higher surface conductivity than the other groups, whereas the reduced lateral conductivity of Ta/H-PEEK indicates that the discrete TaOₓ nanoarray did not form a continuous in-plane conductive pathway.

**Figure S8**. Alamar Blue fluorescence signals for bacterial viability/proliferation, n = 3.

Figure S8 shows the Alamar Blue assay results used to quantify bacterial metabolic activity/viability after incubation with the different sample groups and relevant controls. The fluorescence values provide a quantitative comparison of bacterial activity across conditions, complementing the agar plate and CFU-based antibacterial readouts. The figure is included to support the antibacterial evaluation by offering an independent, metabolism-associated metric that tracks bacterial proliferation/viability after exposure to each surface treatment.

**Figure S9.** Agar plate images and colony comparison for PP, H-PP, Ta-PP, and Ta/H-PP after incubation with bacteria.

Figure S9 provides representative agar plate images and colony comparison for PP-based substrates (PP, H-PP, Ta-PP, and Ta/H-PP), extending the same surface design strategy to a different polymer substrate. The plate images directly visualize differences in colony formation among groups under the same culture conditions. By showing the colony burden across PP, plasma-treated PP, Ta-coated PP, and the combined Ta/H-PP configuration, this figure serves as a substrate-transfer validation dataset and enables side-by-side visual comparison of bacterial colony formation for the PP series.

**Figure S10.** Hemolysis evaluation of PEEK, H-PEEK, Ta-PEEK, and Ta/H-PEEK. Representative photographs of erythrocyte suspensions after centrifugation and quantitative hemolysis ratios of different groups, n = 3.

Figure S10 shows the hemolysis evaluation of different PEEK-based surfaces. The positive control showed obvious hemolysis, whereas the negative control and all sample groups showed clear supernatants after centrifugation. Compared with the negative control, all sample groups showed lower hemolysis ratios, indicating good blood compatibility under the tested conditions.
